# Transcription factor cAMP response element modulator (Crem) restrains Pdgf-dependent proliferation of vascular smooth muscle cells in mice

**DOI:** 10.1007/s00424-014-1652-6

**Published:** 2014-11-27

**Authors:** M. D. Seidl, A. K. Steingräber, C. T. Wolf, T. M. H. Sur, I. Hildebrandt, A. Witten, M. Stoll, J. W. Fischer, W. Schmitz, F. U. Müller

**Affiliations:** 1 Institute for Pharmacology and Toxicology, University of Münster, Domagkstr. 12, D-48149 Münster, Germany; 2 Institute for Human Genetics, Genetic Epidemiology, University of Münster, D-48129 Münster, Germany; 3 Institute for Pharmacology and Clinical Pharmacology, Düsseldorf University Hospital, D-40225 Düsseldorf, Germany

**Keywords:** Vascular tone, Neointima, Proliferation, Crem, CRE-mediated transcription

## Abstract

Transcription factors of the cAMP response element-binding protein (Creb)/cAMP response element modulator (Crem) family were linked to the switch from a contractile to a proliferating phenotype in vascular smooth muscle cells (VSMCs). Here, we analyzed the vascular function of Crem in mice with a global inactivation of Crem (Crem^−/−^). CRE-mediated transcriptional activity was enhanced in primary Crem^−/−^ VSMCs under nonstimulated conditions and under stimulation with Forskolin and platelet-derived growth factor (Pdgf) whereas stimulation with nitric oxide or cGMP showed no effect. This elevated CRE-mediated transcriptional activity as a result of Crem inactivation did not alter aortic contractility or fractions of proliferating or apoptotic aortic VSMCs in situ, and no impact of Crem inactivation on the development of atherosclerotic plaques was observed. Crem^−/−^ mice exhibited an increased neointima formation after carotid ligation associated with an increased proliferation of VSMCs in the carotid media. Pdgf-stimulated proliferation of primary aortic Crem^−/−^ VSMCs was increased along with an upregulation of messenger RNA (mRNA) levels of Pdgf receptor, alpha polypeptide (*Pdgfra*), cyclophilin A (*Ppia*), the regulator of G-protein signaling 5 (*Rgs5*), and Rho GTPase-activating protein 12 (*Arhgap12*). Taken together, our data reveal the inhibition of Pdgf-stimulated proliferation of VSMCs by repressing the Pdgf-stimulated CRE-mediated transcriptional activation as the predominant function of Crem in mouse vasculature suggesting an important role of Crem in vasculoproliferative diseases.

**Electronic supplementary material:**

The online version of this article (doi:10.1007/s00424-014-1652-6) contains supplementary material, which is available to authorized users.

## Introduction

cAMP response element-binding protein (Creb) and cAMP response element modulator (Crem) are structurally related transcription factors regulating the transcription of multiple genes in response to cAMP, cGMP, and other second messengers [33, 32]. Both transcription factors exhibit a notable functional diversity through the existence of splice variants and the formation of homodimers or heterodimers binding to the cAMP response element (CRE; TGACGTCA and variants thereof) in the promoter regions of their target genes [17, 25]. The phosphorylation of Creb at Ser-133 by the cAMP-dependent protein kinase A (PKA) represents an important mechanism of CRE-mediated transcriptional activation. In contrast, a number of Crem variants, particularly small isoforms including inducible cAMP early repressor (Icer) and small Icer (smIcer), suppress CRE-mediated transcriptional activation [38, 26, 7]. These repressors contain the DNA-binding domain enabling them to bind to the CRE; however, they lack functional domains responsible for transactivation or the inducibility by kinases [26].

Cyclic AMP promotes quiescence and maintenance in vascular smooth muscle cells (VSMCs) and the β-adrenoceptor, and cAMP-dependent activation of PKA is important to sustain their contractile function under normal conditions [35]. Creb is an important downstream target of PKA, and changes in Creb phosphorylation or function are correlated with the regulation of cell proliferation [18, 41], apoptosis [42], migration [15, 29], VSMC hypertrophy [8], or vascular resistance [23]. Elevated cAMP levels inhibit platelet-derived growth factor (Pdgf)-stimulated VSMC growth by PKA/Creb-dependent induction of transformation-related protein 53 (p53) and cyclin-dependent kinase inhibitor 1A (p21) and by inhibition of mitogen-activated protein kinase (MAPK) signaling [2, 11]. Several studies showed the relevance of Creb for the regulation of vascular quiescence and the development of vasculoproliferative diseases [35, 14]. Schauer et al. reported that downregulation of Creb is a common pathological response to vascular injury and seems to contribute to plaque progression [37]. It is suggested that most Crem isoforms (including Icer and smIcer) act as repressors of CRE-dependent gene transcription. Therefore, Crem might play an important role as a counterpart of Creb in the vasculature.

Aldosterone-induced impaired vascular contractility reportedly goes along with diminished glucose-6 phosphate dehydrogenase expression due to increased Crem levels [22], and a transient overexpression of Icer inhibited neointima formation in balloon-injured rat carotid artery [28]. However, the physiological role of Crem in the intact vascular system is fragmentary and has not yet been systematically studied in knockout models.

In order to elucidate the general role of Crem in the vasculature, we investigated the CRE-mediated transcriptional activation in response to important stimuli in the vasculature, namely nitric oxide (NO), cAMP, cGMP, and Pdgf in Crem knockout mice (Crem^−/−^) and wild-type (Crem^+/+^) controls. Since Pdgf and cAMP stimulation increased CRE-mediated gene expression in Crem^−/−^ vs. Crem^+/+^ VSMCs, we investigated the relevance of enhanced CRE-mediated transcription by Crem inactivation under physiological and pathological conditions in the vasculature. In particular, the impact of Crem inactivation on vascular contractility, plaque development after high-fat diet, and neointima formation after vascular injury by carotid ligation was assessed. Moreover, we analyzed apoptosis and proliferation rates of aortic VSMCs and identified possible target genes of Crem relevant in this context.

## Material and methods

For detailed description of cell culture; transient transfection; histological, histochemical, and immunofluorescence imaging; and quantitative real-time PCR, see the expanded material and methods part in the online supplement.

### Experimental animals and materials

The generation of Crem mutant mice lacking the DNA-binding domains of the *Crem* gene was published previously [3]. We thank Dr. G. Schütz, DKFZ Heidelberg, Germany, for providing Crem^−/−^ mice. Crem^−/−^ and Crem^+/+^ mice originated from the same mouse colony which has been continued for more than ten generations on the same mixed background (*129Sv*:*C57*/*Bl6*). All animal experimentation was performed in accordance with local animal welfare authorities and approved by the LANUV-Regional Authority for Nature, Environment and Consumer protection in North Rhine-Westphalia, Germany (permit number: 8.87-51.04.20.09.386 or 84.02.04.2011.A179). Surgery was performed under isoflurane-nitrous oxide anesthesia, and every effort was made to minimize suffering. All chemicals were purchased from Sigma (Sigma-Aldrich Chemie GmbH, Steinheim, Germany) as not stated otherwise.

### Analysis of aortic reactivity

Aortic vascular tone was measured in male Crem^−/−^ and Crem^+/+^ mice of 17–18 weeks. Preparation and initial treatment of thoracic aortae as well as mounting to the dual-wire myograph system (model 410A; J.P. Trading, Aarhus, Denmark) were described before [19]. After 45 min of equilibration in a 10-ml bath of physiologic salt solution (in mmol/l, 118 NaCl, 25 NaHCO_3_, 2.5 CaCl_2_, 4.7 KCl, 1.2 KH_2_PO_4_, 1.2 MgSO_4_, 5.5 glucose, and 0.026 ethylene diamine tetraacetate) at 37 °C, aortic rings were stretched to a passive resting tension of 17.5 mN, followed by several constrictions induced by a high-potassium solution (K-PSS) composed of (in mmol/l) the following: 25 NaHCO_3_, 2.5 CaCl_2_, 122.7 KCl, 1.2 KH_2_PO_4_, 1.2 MgSO_4_, 5.5 glucose, and 0.026 ethylene diamine tetraacetate. Vasoconstriction was measured after stimulation with the α-adrenoceptor agonist phenylephrine (PE) or prostaglandin F_2α_. Vasorelaxation was studied after precontraction with 1 μmol/l PE and stimulation with the m-cholinoceptor agonist carbachol, the β-adrenoceptor agonist isoproterenol, or with the nitrogen oxide donor sodium nitroprusside. Vasoconstrictions and relaxations (Fig. 3a, c–e) were determined as first effects after K^+^ contractions except prostaglandin F_2α_ (PGF_2α_, second effect after PE contraction experiments) followed by an injection of 1 μmol/l carbachol to test endothelial response (exclusion criterion less than 70 % relaxation). PE-induced vasoconstriction and precontractions for carbachol and sodium nitroprusside were performed in the presence of 1 μmol/l propranolol to exclude possible β-adrenergic effects of PE. For the separate endothelium-denuded experiments, the endothelium was removed by carefully rubbing a wire on the inner side of the aortic rings. The successful endothelium denudation was checked by the relaxation response before and after denudation by 10^−6^ M acetylcholine stimulation after norepinephrine (10^−6^ M) contraction. All drugs were directly added to the bath. For treatment of mice with isoprenaline, osmotic mini-pumps (model 2001, Alzet, Cupertino, USA) were implanted in mice for continuous application of 10 mg/kg body weight/day of isoprenaline (dl-isoprenaline hydrochloride) for 7 days according to the manufacturer’s instructions.

### Mouse carotid ligation model and sham operation

Carotid artery ligation model was performed with male Crem^−/−^ and Crem^+/+^ mice as a modification of the model published by Kumar et al. [20, 21]. Mice were anesthetized by intraperitoneal injection of avertin solution (tribromoethanol 0.36 mg/g body weight with 2-methyl-2-butanol 0.18 mg/g body weight). A midline neck incision was made to expose the left and right common carotid arteries. Sham operation was performed on the left artery as a contralateral control, before the right carotid artery was ligated by a 5-0 propylene suture proximal to the carotid bifurcation. After 3 weeks of recovery, mice were sacrificed and the carotid arteries were excised and fixated overnight in neutral buffered formaldehyde (4 %). Tissues were dehydrated and embedded in paraffin as described above. Series of eight 5-μm sections were prepared on glass slides of the right and left arteries. For the right ligated arteries, carotid sections in a proximal distance to ligation of 0, 200, 400, 700, and 1000 μm were collected and stained for morphometric evaluation with resorcin-fuchsin and nuclear fast red. For the sham-ligated left carotid arteries, morphometric values were obtained from three different slides and calculated as a mean for each vessel. For immunohistochemical analysis, sections of the right carotid artery were investigated in a proximal distance to ligation of 0–200 μm.

### Statistics

If not stated otherwise, data are expressed as mean values ± standard error of the mean (SEM) with *n* indicating the number of independent experiments. In cell culture experiments, the number of independent transfections and the number of separate cells isolations are indicated. For statistical evaluation, two-way ANOVA and Tukey post hoc tests were used to compare multiple groups. For comparison of two groups, Student’s *t* test was conducted. Statistical differences were considered as significant at *p* < 0.05. All statistical analyses were performed using SigmaPlot (version 11.2; Systat Software, Erkrath, Germany) except the statistical analysis of RT-PCR results and calculation of relative expression ratios which were conducted by the relative expression software tool (REST© Version 2.07) [30, 44].

## Results

### Increased CRE-dependent transcriptional activity in VSMCs from Crem^−/−^ mice after stimulation with cAMP or Pdgf

**Fig. 1:**
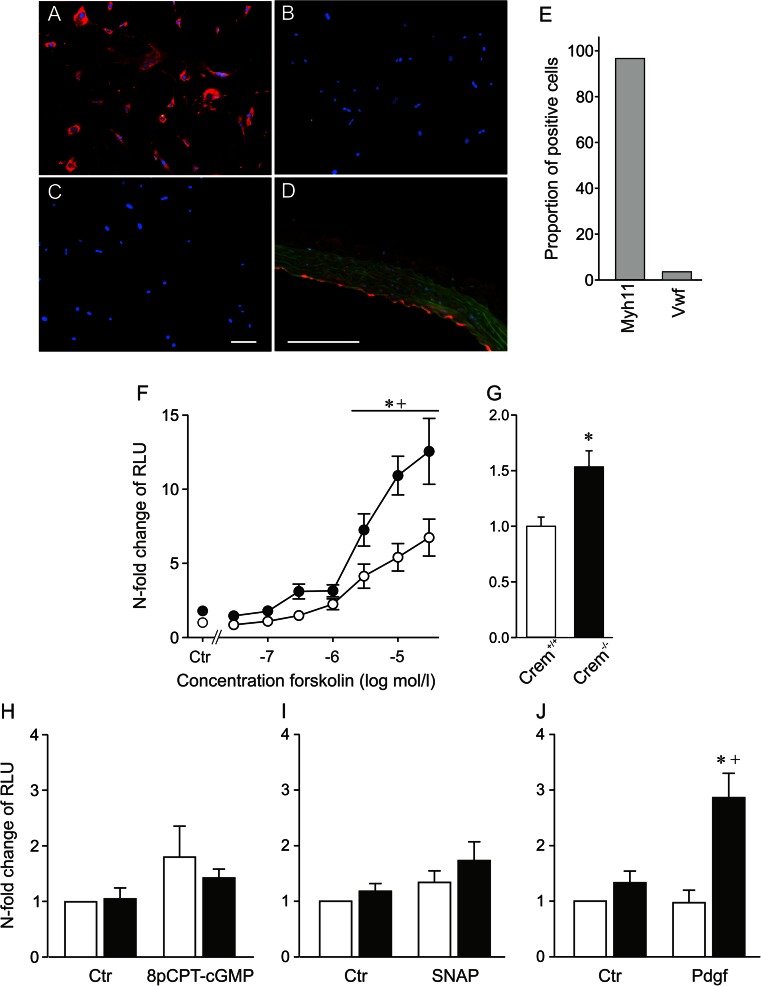
Characterization of VSMC culture by immunofluorescence staining (**a**–**e**). Photographs of isolated VSMCs incubated with an antibody against the **a** smooth muscle myosin heavy chain (Myh11), **b** Von Willebrand factor (Vwf), **c** the secondary antibody control without primary antibody, and **d** positive control of the Vwf antibody in aortic sections; note the positive red staining of the endothelium. **e** Quantification of the proportion of stained cells based on 350 analyzed cells revealed over 95 % positive staining of the cells by the VSMC marker Myh11; *scale bar* 100 μm. **f**–**j** CRE-mediated transcriptional activation of isolated VSMCs from Crem^+/+^ (*white symbols*) and Crem^−/−^ (*black symbols*) mice. VSMCs were transiently transfected with a CRE-controlled luciferase reporter gene construct and treated with activators of different signaling cascades or solvent controls (*Ctr*). **f** Forskolin (in DMSO, *n* = 8/6 meaning eight independent transfections from six isolations, for 12 h; Ctr: *n* = 15–18/6), **h** 10^−4^ M 8-pCPT-cGMP (in H_2_O, *n* = 6/6, for 9 h), **i** 10^−4^ M SNAP (in H_2_O, *n* = 7/7, for 9 h), and **j** 7.5 ng/ml Pdgf (in H_2_O, *n* = 5/5, for 9 h). Data were presented relative to the mean of Crem^+/+^ Ctr, which was set to 1. Note the elevated CRE-dependent transcriptional activity under stimulation with 3 × 10^−8^–3 × 10^−5^ M Forskolin (**f**) and Pdgf (**j**) in VSMCs of Crem^−/−^ mice. Independent analysis of untreated transfected VSMCs (**g**) revealed a 1.5-fold increase in VSMCs from Crem^−/−^ vs. Crem^+/+^ mice (*n* = 51–54/3). *Asterisk* (*) denotes *p* < 0.05 vs. Crem^+/+^, a *cross* (+) denotes *p* < 0.05 vs. control

At first, we tested the effect of Crem inactivation on CRE-mediated transcriptional activation in isolated primary aortic VSMCs. Staining of these cells revealed a proportion >95 % of the smooth muscle marker smooth muscle myosin heavy chain (Myh11), while the marker of endothelial cells Von Willebrand factor (Vwf) was detectable in less than 4 % of the cells (Fig. 1a–e). CRE-dependent transcriptional activity was measured after transient transfection of VSMCs with a CRE-controlled reporter gene plasmid and subsequent stimulation with activators of the cAMP- (Forskolin, a direct receptor-independent activator of the adenylyl cyclase) or cGMP-dependent (8-pCPT-cGMP and *S*-nitroso-*N*-acetylpenicillamine (SNAP)) signaling cascades or in response to the growth factor Pdgf. Cells were stimulated with different concentrations of Forskolin (3 × 10^−8^–3 × 10^−5^ mol/l), or DMSO (1 mmol/l) as solvent control. (Fig. 1f). Forskolin increased the CRE-controlled promoter activity in a concentration-dependent manner in both groups. However, Crem inactivation led to an enhanced induction of CRE-controlled transcriptional activity after stimulation with 3 × 10^−6^ to 3 × 10^−5^ mol/l Forskolin; nonstimulated VSMCs of Crem^−/−^ mice showed a significant 1.5-fold increase in the CRE-controlled transcriptional activity (Fig. 1g; *p* < 0.05). Hence, the inactivation of Crem in Crem^−/−^ mice was associated with an increased CRE-dependent transcriptional activity both in response to Forskolin and under basal conditions in aortic VSMCs. Stimulation with the cGMP analogon 8-pCPT-cGMP (9 h; 10^−4^ M) or with the NO donator SNAP (9 h; 10^−4^ M) did not reveal an increase in CRE-mediated transcriptional activity versus nonstimulated control conditions in both genotypes (Fig. 1h, i). Stimulation with the growth factor Pdgf (9 h; 7.5 ng/ml) exclusively increased the CRE-mediated gene expression in Crem^−/−^ VSMCs while Pdgf had no effect in Crem^+/+^ cells, indicating that Crem can completely block the impact of Pdgf on CRE-dependent gene expression (Fig. 1j).

### No morphological differences and an unaltered rate of apoptotic or proliferating aortic VSMCs in the aorta of Crem^−/−^ mice

**Fig. 2:**
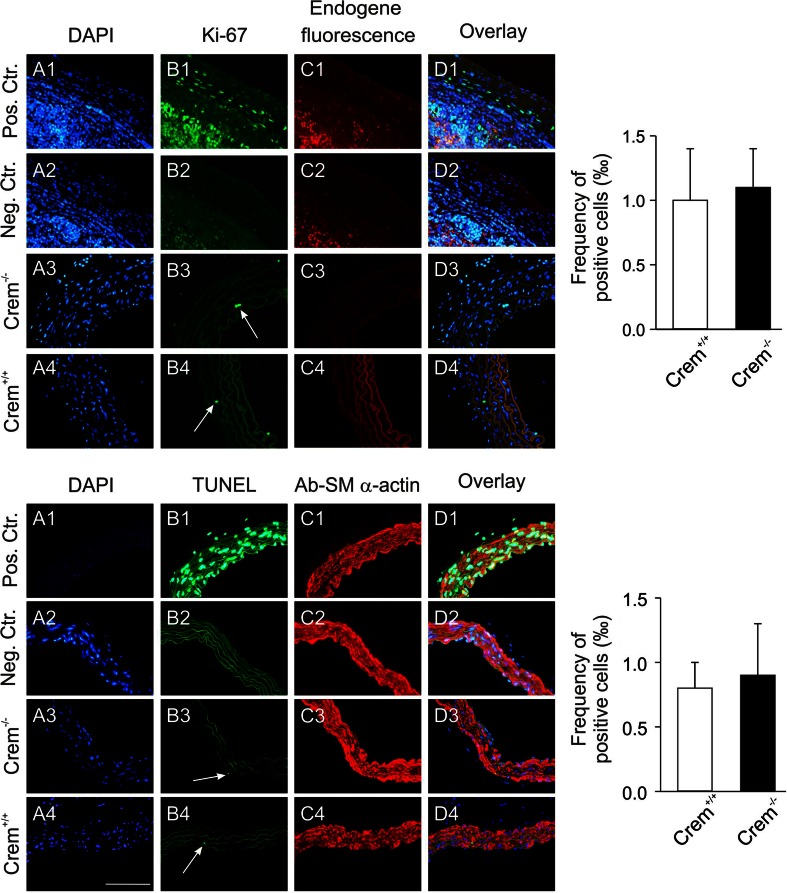
Detection of proliferating VSMCs (*top*) in aortic sections from Crem^−/−^ and Crem^+/+^ mice (*A3*–*D3* and *A4*–*D4*). Photomicrographs show the following: DAPI cell nuclei staining (*A1*–*4*), proliferating VSMCs detected by a Ki-67 antibody (*B1*–*4*; *white arrows*), the endogenous fluorescence (*C1*–*4*), and the overlay (*D1*–*4*). As a positive control, spleen tissue sections with a high proliferation rate were stained with the Ki-67 antibody (*A1*–*D1*) and were incubated as a negative control without the first Ki-67 antibody (*A2*–*D2*) (*bottom*). Detection of apoptotic VSMCs in aortic sections from Crem^+/+^ and Crem^−/−^ mice (*A3*–*D3* and *A4*–*D4*). Photomicrographs show the following: the cell nuclei stained with DAPI (*A1*–*4*), the apoptotic VSMCs detected by a TUNEL assay (*B1*–*4*; *white arrows*), VSMCs visualized by a smooth muscle actin-specific antibody (*C1*–*4*), and the overlay (*D1*–*4*). As a positive control, aortic sections were treated with DNase I (*A1*–*D1*), and as a negative control, sections were treated without enzyme (*A2*–*D2*). Statistical analysis of the frequency of apoptotic cells in the aortic sections of Crem^+/+^ (*white*) and Crem^−/−^ (*black*) mice revealed no significant differences in the fraction of proliferating or apoptotic VSMCs in the aorta (*right*); *n* = 3 based on 5960 ± 550 cells for detection of proliferating cells and 6030 ± 220 cells for detection of apoptotic cells in each group, respectively

The increased CRE-mediated transcriptional activation, in particular under basal, nonstimulated conditions (Fig 1g) in Crem^−/−^ mice raised the question whether inactivation of Crem is associated with functional alterations in the mouse vasculature. However, histological analysis of aortae from Crem^−/−^ mice did not show any differences in comparison to Crem^+/+^ mice. There was no change in the thickness of the media (Crem^+/+^, 53 ± 1 μM, *n* = 3; Crem^−/−^, 54 ± 4 μM, *n* = 3). In order to assess possible effects of Crem inactivation on important functions of aortic smooth muscle cells, we studied the impact of Crem inactivation on the fraction of apoptotic and proliferating aortic VSMCs. There were no significant differences between Crem^−/−^ and Crem^+/+^ mice regarding both the basal proliferation rate and the proportion of apoptotic cells of VSMCs (Fig. 2).

### Unaltered regulation of aortic vascular tone in Crem^−/−^ mice

**Fig. 3:**
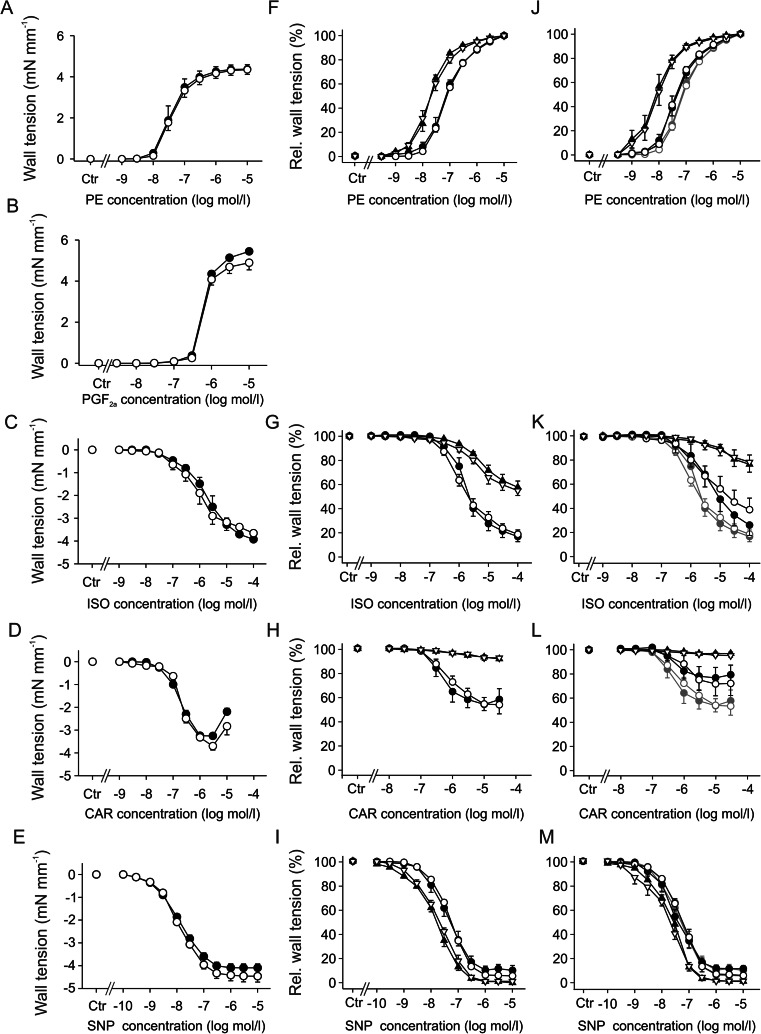
Cumulative drug-induced contractions and relaxations in wall tension of aortic rings (**a**–**e**), relative wall tensions after denudation (*triangles*) compared to intact aortae (*circle*) without (**f**–**i**) and with 1 week of chronic isoproterenol treatment (**j**–**m**) in Crem^+/+^ (*open symbols*) and Crem^−/−^ (*filled symbols*) male mice. *Curves in dark gray* (**j**–**m**) show data of untreated aortae (**f**–**i**). The vascular tone was measured in response to stimulation with the α-adrenoceptor agonist phenylephrine (**a**, **f**, **j**; *PE*, *n* = 4–6) and prostaglandin F_2α_ (**b**; *PGF*_*2α*_, Crem^+/+^ *n* = 4, Crem^−/−^ *n* = 3). Relaxations were induced after preconstriction with PE (1 μmol/l) by the β-adrenoceptor agonist isoproterenol (**c**, **g**, **k**; *ISO*; *n* = 4–6), the muscarinic receptor agonist carbachol (**d**, **h**, **l**; *CAR*; *n* = 5–8), and the nitric oxide donor sodium nitroprusside (**e**, **i**, **m**; *SNP*; *n* = 4–9). No significant differences between Crem^+/+^ and Crem^−/−^ mice were observed

Vascular tone was studied in aortic rings from Crem^−/−^ and Crem^+/+^ mice in a wired myograph system. Concentration-dependent vasoconstriction was measured under stimulation with the α_1_-adrenoceptor agonist phenylephrine (PE, Fig. 3a) or with prostaglandin F_2α_ (PGF_2a_, Fig. 3b). Vasodilatation was studied after precontraction with 1 μmol/l PE and application of increasing concentrations of the β-adrenoceptor agonist isoproterenol (ISO, Fig. 3c). NO-dependent vasodilatation was measured under stimulation with both the endothelium-dependent NO-releasing m-cholinoceptor agonist carbachol (CAR, Fig. 3d) or the endothelium-independent NO donor sodium nitroprusside (SNP, Fig. 3e). Overall, there were no significant differences between groups, neither in maximum or in minimum wall tension of contraction or relaxation nor in the half maximal effective concentration (EC_50_)/half maximal inhibitory concentration (IC_50_) values (see supplemental Fig. S1). To exclude endothelium-dependent effects, aortic contractility was also studied in denuded aortae after PE, ISO, CAR, and SNP stimulation (Fig. 3f–i). Moreover, contractility was measured after 7 days of isoproterenol administration in intact and denudated aortae (Fig. 3j–m). Denudation showed a marked impact on aortic contractility. PE showed a higher potency reflected by a left shift of the dose-response curve. The relaxation after ISO or CAR treatment was markedly impaired after denudation, while SNP treatment showed a slight increase in efficiency and potency. Isoproterenol treatment showed a similar but less pronounced effect on PE, ISO, and CAR dose-response curves which could be superimposed by denudation, while the chronic isoproterenol treatment had no effect on the SNP-stimulated relaxation. However, both denudation of aortic rings as well as isoproterenol treatment and their combination showed no difference between Crem^−/−^ and Crem^+/+^ mice.

### Unaltered plaque development in Crem^−/−^ × ApoE^−/−^ mice

After analysis of basal morphological and physiological functions, we investigated the impact of Crem inactivation in atherosclerotic plaque development in aortae on an *ApoE* knockout (ApoE^−/−^) background and in a model of vascular injury by carotid ligation. Red oil O staining of aortae revealed no difference in plaque area in Crem^−/−^ × ApoE^−/−^ compared to Crem^+/+^ × ApoE^−/−^ mice after 20 weeks of high-fat diet, while a slight 7 % increase after 30 weeks of high-fat diet was observed. The control groups showed small red oil O-positive areas with no differences between genotypes after 30 weeks of standard diet (supplemental Fig. S2A, B). A detailed analysis of the root of the aortic arch showed no difference in the main plaque area and the area-positive macrophage staining (supplemental Fig. S2C, D). Moreover, no differences were found in the proliferation rate of VSMCs in the media (Crem^+/+^ × ApoE^−/−^ 0.48 ± 0.17 vs. Crem^−/−^ × ApoE^−/−^ 0.32 ± 0.17 % VSMCs) and in the proliferation rate of all cell types in the plaques (Crem^+/+^ × ApoE^−/−^ 4.1 ± 0.5 vs. Crem^−/−^ × ApoE^−/−^ 3.7 ± 0.8 % VSMCs; see supplemental Fig. S2F). Analysis of cell content showed a comparable cell density in the media (Crem^+/+^ × ApoE^−/−^ 3844 ± 453 vs. Crem^−/−^ × ApoE^−/−^ 3120 ± 228 cells/mm^2^) and the plaques (Crem^+/+^ × ApoE^−/−^ 3190 ± 244 vs. Crem^−/−^ × ApoE^−/−^ 3331 ± 389 cells/mm^2^) between the genotypes. Measurement of triglycerides, high- and low-density lipoprotein (HDL and LDL, respectively) and cholesterol revealed no differences between the genotypes (see supplemental Fig. S3). Overall inactivation of Crem had a minor impact on the progression of plaque development.

### Increased neointima formation after ligation of carotids in Crem^−/−^ mice

**Fig. 4:**
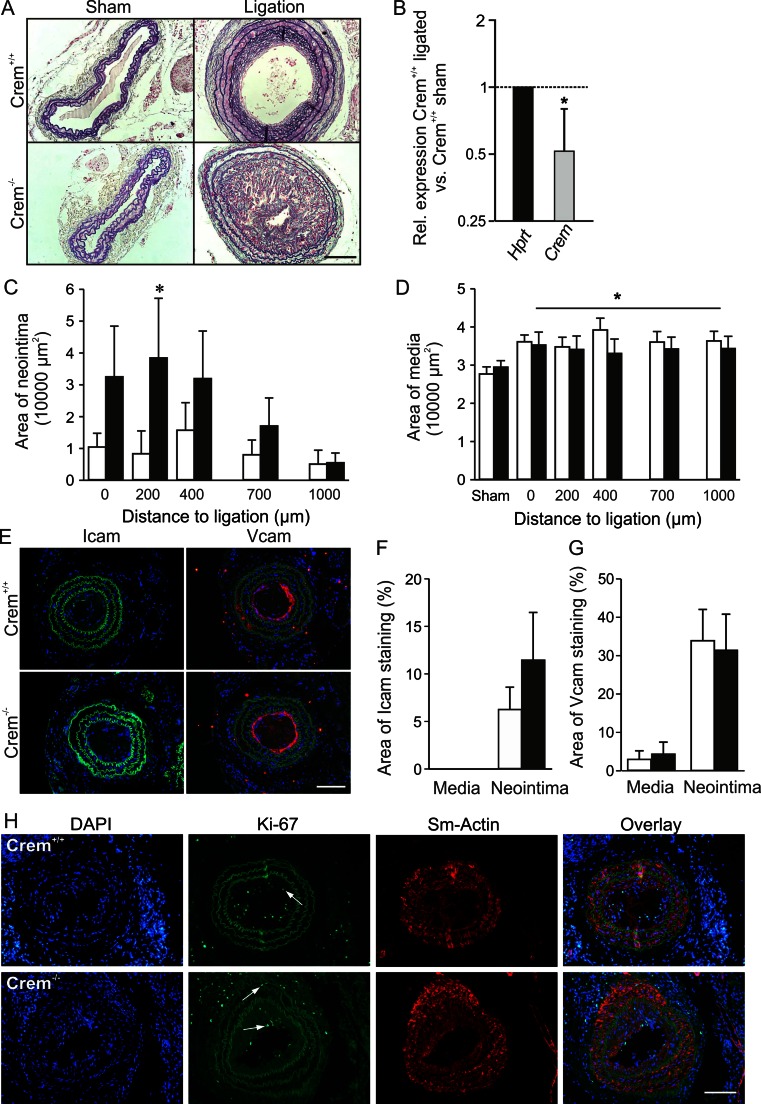
Analysis of neointima formation of the right carotid artery 3 weeks after ligation. **a** Resorcin-fuchsine and nuclear fast red staining of the cross sections of the ligated right and sham-operated left carotid artery from Crem^+/+^ and Crem^−/−^ mice. **b** Downregulation of relative *Crem* mRNA levels in Crem^+/+^ sham (*n* = 6) and ligated carotids (*n* = 4). Quantification of neointima (**c**) and media area (**d**) in Crem^+/+^ (*white*, *n* = 10) and Crem^−/−^ mice (*black*, *n* = 9). Note the severe increase in neointima formation in the Crem^−/−^ mice. **e** Icam1- and Vcam1-specific immunofluorescent staining of carotid cross sections of ligated carotids from Crem^+/+^ and Crem^−/−^ mice. Quantification of Icam1- (**f**) and Vcam1 (**g**)-stained area showed no differences between the genotypes. *p < 0.05 vs. Crem^+/+^ (post hoc tests, two-way ANOVA). **h** Detection of proliferating VSMCs in the cross sections of ligated carotids. Photomicrographs show the following: the cell nuclei stained with DAPI, proliferating cells (VSMCs) detected by a Ki-67 antibody (*white arrows*), visualization of VSMCs with a smooth muscle actin-specific antibody, and the overlay. Crem^−/−^ VSMCs showed an increase in the percentage of proliferating VSMCs in the media but not in the neointima (for details, see text). *Scale bars* = 100 μm

Crem^−/−^ mice showed a severe increase in neointima formation (Fig. 4a, c) when compared to Crem^+/+^ mice, while elevation of media thickness compared to the sham controls remained equal between the genotypes (Fig. 4d). Quantification of relative *Crem* messenger RNA (mRNA) levels revealed a 48 % decrease in sham versus ligated WT carotids (Fig. 4b). Furthermore, no difference in the expression of intercellular adhesion molecule 1 (Icam1) and vascular cell adhesion molecule 1 (Vcam1) was observed between genotypes (Fig. 4d–f). The increased neointima formation in Crem^−/−^ mice was accompanied by a significant increase of proliferating VSMCs in the media (Crem^+/+^ 0.11 ± 0.06 vs. Crem^−/−^ 0.79 ± 0.26 % VSMCs) but not in the neointima (Crem^+/+^ 4.5 ± 2.5 vs. Crem^−/−^ 1.9 ± 0.7 % VSMCs; see Fig. 4g). Separate analysis of cell content showed a comparable cell density in the media (Crem^+/+^ 4451 ± 259 vs. Crem^−/−^ 4931 ± 195 cells/mm^2^) and the neointima (Crem^+/+^ 6374 ± 488 vs. Crem^−/−^ 6648 ± 1484 cells/mm^2^) between genotypes.

### Increased fraction of proliferating primary VSMCs but unaltered fraction of apoptotic VSMCs in Crem^−/−^ mice

**Fig. 5:**
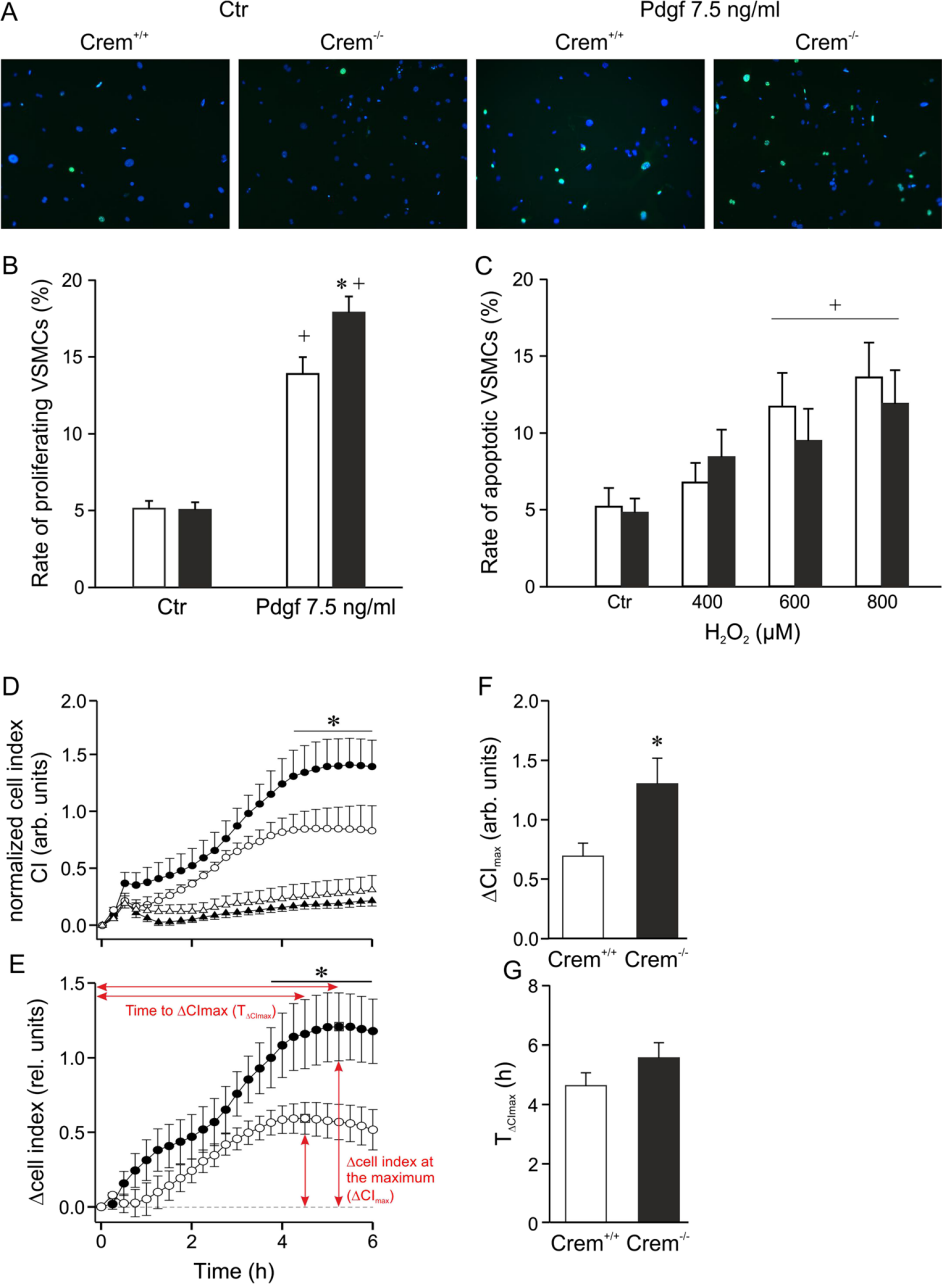
Detection of proliferating and apoptotic cells in primary isolated VSMCs from Crem^+/+^ (*white bars*) and Crem^−/−^ (*black bars*) mice. **a** Immunofluorescence images of VSMCs stained with DAPI and a Ki-67 antibody to detect proliferating cells after stimulation with Pdgf (7.5 ng/ml, 24 h) compared to control conditions without stimulation (*Ctr*). **b** Quantitative analysis showed an increase in the percentage of proliferating VSMCs in Crem^−/−^ vs. Crem^+/+^ VSMCs in the Pdgf-treated groups (*n* = 14/5). **c** Percentage frequency of apoptotic VSMCs was detected by TUNEL assay after 24-h stimulation with increasing concentrations of hydrogen peroxide. No differences between the genotypes were observed (*n* = 10/6). **d** Impedance-based real-time proliferation curves of vehicle-treated (*triangle*) and Pdgf (7.5 ng/ml)-treated VSMCs (*circle*) of Crem^−/−^ (*n* = 8; *black*) and Crem^+/+^ VSMCs (*n* = 8; *white*) normalized to the time point before Pdgf treatment (**e**). The differential proliferation curves of Pdgf-treated minus Pdgf-untreated VSMCs and the calculation of the **f** maximum cell index (*ΔCI*_*max*_; *square*) and the **g** time to the maximum (*T*_*ΔCImax*_) are shown (all *n* = 8). Note the increased proliferation rate in Pdgf-stimulated Crem^−/−^ compared to Crem^+/+^ VSMCs, represented by the increased cell index of proliferation curves and the higher ΔCI_max_ values, while the time to ΔCI_max_ was not changed. *Asterisk* (*) denotes *p* < 0.05 vs. Crem^+/+^, and the *cross* (+) denotes *p* < 0.05 vs. control conditions. For statistical analysis of proliferation curves, a two-way repeated measures ANOVA and Tukey post hoc tests were used

Since an elevated proliferation rate of VSMCs was observed in the ligated carotids, we studied effects of Crem inactivation in primary VSMCs both under basal conditions and under stimulation of proliferation with Pdgf. There was no difference in the proliferation under nonstimulated control conditions. Stimulation with Pdgf (7.5 ng/ml) for 24 h increased the number of proliferating cells in both genotypes (Fig. 5a, b). Under this condition, the proportion of proliferating cells was 1.3-fold higher in Crem^−/−^ vs. Crem^+/+^ VSMCs. Furthermore, we studied the rate of apoptotic VSMCs under basal conditions and after triggering apoptosis with H_2_O_2_. H_2_O_2_ significantly increased the rate of TUNEL-positive cells in a concentration-dependent manner in both groups (Fig. 5c). However, there were no differences between genotypes. To substantiate the finding of increased VSMC proliferation rate in Crem^−/−^ mice, we measured cell growth in an impedance-based Real-Time Cell Analyzer (RCTA). Again, cell growth of Crem^−/−^ VSMCs was elevated compared to *Crem* ^+/+^ under stimulation with Pdgf, while proliferation rate under basal condition was unaltered (Fig. 5d). This was also represented by the differential proliferation curves of Pdgf-treated and Pdgf-untreated cells (Fig. 5e) and the 2-fold higher maximum cell index (CI), while the time point of the maximum CI was not altered (Fig. 5f, g).

To identify genes which are differentially regulated between the Pdgf-stimulated VSMCs of Crem^−/−^ and Crem^+/+^ mice, a microarray analysis was performed (data not shown). Verification of putative-regulated genes by quantitative real-time PCR analysis (Table 1, *n* = 5) revealed an upregulation of Rho GTPase-activating protein 12 (*Arhgap12*), cyclophilin A (*Ppia*), the regulator of G-protein signaling 5 (*Rgs5*), and the Pdgf receptor, alpha polypeptide (*Pdgfra*) mRNA in Pdgf-stimulated VSMCs from Crem^−/−^ vs. Crem^+/+^ mice. Other genes which tended to be upregulated comprise the fibroblast growth factor 18 (*Fgf18*), angiotensin II, type I receptor-associated protein (*Agtrap*), and TAF12 RNA polymerase II, TATA box-binding protein (TBP)-associated factor (*Taf12*).

**Table 1:** Expression analysis of selected genes in VSMCs of Crem^−/−^ and Crem^+/+^ mice stimulated with 7.5 ng/ml Pdgf-BB by quantitative real-time PCR

Gene	Relative expression	Standard error	*p* value
*Hprt*	1.054		
*Ywhaz*	0.949		
*Ppia*	1.450	1.13–1.87	0.004 up
*Arhgap12*	1.675	1.29–2.24	0.006 up
*Rgs5*	2.562	1.27–4.81	0.009 up
*Pdgfra*	1.568	1.09–2.55	0.026 up
*Taf12*	1.458	0.93–2.23	0.052
*Fgf18*	1.716	0.92–2.81	0.054
*Agtrap*	5.125	0.73–20.88	0.062

Relative expression ratios, standard error, and statistical analysis were calculated by the relative expression software tool (REST© Version 2.07). An upregulation indicates an elevated amount of mRNA in the Crem^−/−^ VSMCs (*n* = 5)

Rgs5 mRNA levels were also upregulated in untreated aortae of Crem^−/−^ compared to Crem^+/+^ mice, while the mRNA levels of the other genes were unaltered (Table 2, *n* = 11–12).

**Table 2:** Expression analysis of selected genes in the aorta of Crem^−/−^ and Crem^+/+^ mice by quantitative real-time PCR

Gene	Relative expression	Standard error	*p* value
*Hprt*	1.000		
*Ppia*	1.102	0.74–1.67	0.454
*Arhgap12*	1.049	0.42–2.50	0.858
*Rgs5*	1.909	0.87–4.11	0.011 up
*Pdgfra*	0.984	0.52–1.85	0.934
*Taf12*	0.945	0.53–1.76	0.779
*Fgf18*	0.801	0.44–1.51	0.245
*Agtrap*	1.043	0.43–2.45	0.875

Relative expression ratios, standard error, and statistical analysis were calculated by the relative expression software tool (REST© Version 2.07). An upregulation indicates an elevated amount of mRNA in the Crem^−/−^ aortae (*n* = 11–12)

## Discussion

In this study, we systematically analyzed the relevance of the cAMP-mediated transcription factor Crem in the vasculature under physiological and pathophysiological conditions. Main results of the functional inactivation of Crem comprise the following: (i) an increased CRE-mediated transcriptional activation under basal conditions and in response to stimulation with Forskolin and Pdgf in isolated primary VSMCs. (ii) The enhanced CRE-mediated transcription was not associated which a major impact in the following: the physiology of aortic VSMCs under basal conditions, aortic contractility, and plaque development after high-fat diet. (iii) Contrary to this inconspicuous phenotype, a severe increase of neointima development after carotid ligation was observed, which was accompanied by an elevated proliferation rate of VSMCs in the media. (iv) In accordance to this finding, an increased amount of proliferating VSMCs under stimulation with Pdgf in isolated VSMCs was observed. Hence, we showed that as a predominant function in VSMCs, Crem represses the Pdgf-activated CRE-mediated gene transcription, which is particularly important for the regulation of proliferation, suggesting an important role of Crem in vasculoproliferative diseases.

To evaluate the functionality of the inactivation of Crem in VSMCs and to exclude possible compensatory effects of other transcription factors acting at CREs, we analyzed the CRE-mediated transcriptional activity in response to Forskolin to stimulate the cAMP-dependent signaling cascade. Such compensatory effects were previously observed in different genetic mouse models [3, 13, 9]. For experiments, isolated VSMCs of low passages (<2) and high purity over 95 % of Myh11-positive cells were used to minimize effects of phenotypic modulation [5] and contamination of other cell types, e.g., endothelial cells. Crem inactivation in VSMCs increased CRE-mediated transcriptional activity, likely as a consequence of a derepressed activity of CREB or other activating transcription factors like activating transcription factor 1 (ATF1). Therefore, Crem isoforms appear to have predominantly inhibitory functions in VSMCs and the increase in CRE-mediated gene expression underscores the functionality of the Crem inactivation and excludes the possibility that a lacking vascular phenotype of Crem^−/−^ mice under basal conditions is due to a full compensation within the Creb/Crem family of transcription factors. Besides, the cAMP-dependent signaling NO and the soluble guanylate cyclase/cGMP signaling cascade have been suggested to phosphorylate Creb either by activation of mitogen-activated kinases or by direct phosphorylation of Creb (for review, see [31]). Steinbicker et al. reported that treatment of rat pulmonary artery cells with the NO donor *S*-nitroso-glutathione induced the Crem isoform Icer by activated CRE-mediated gene expression [40]. These results suggest that NO is a potent regulator of CRE-mediated gene expression. However, using the NO donor SNAP or a cGMP analogon, we did not find an induction or differences of CRE-mediated gene expression in the primary aortic VSMCs, indicating a minor role of NO for CRE-dependent gene expression in murine aortic VSMCs under our experimental conditions. However, stimulation of VSMCs with Pdgf revealed an increase of CRE-mediated gene expression in the Crem^−/−^ VSMCs which was completely blunted in the Crem^+/+^ cells. This indicates that the stimulation with Pdgf activates CRE-mediated gene expression, but this mechanism is controlled by Crem.

Since cAMP-dependent CRE-mediated gene expression was already increased in Crem^−/−^ aortic VSMCs under basal physiological conditions, we first studied the impact of Crem inactivation on aortic contractility, VSMC proliferation, apoptosis, and morphological abnormalities with regard to possible physiological changes. Previous findings by catheterization of the left ventricle showed that Crem^−/−^ mice exhibit a lower left-ventricular blood pressure than Crem^+/+^ animals [27], which could be associated with alterations in the contractility of the vascular system. Moreover, a loss of the Creb isoforms α and Δ was linked to a higher pulmonary vascular resistance [23]. Our analysis revealed that neither the PE- or prostaglandin-mediated vasoconstriction nor the β-adrenergic, muscarinic, or NO-dependent vasorelaxation was different concerning the maximal effects or the EC_50_/IC_50_ in Crem^−/−^ mice versus Crem^+/+^ mice. Furthermore, no differences in contractility of Crem^−/−^ mice versus Crem^+/+^ mice could be detected in endothelium-denuded aortae and in aortae of 1 week isoproterenol-treated animals. Consequently, our data indicate that Crem-derived transcription factors do not play an important role in the regulation of proteins responsible for the regulation of the aortic contractility, on the one hand, in both VSMCs and endothelial cells and, on the other hand, under basal and catecholamine-induced stress conditions reflected by the isoproterenol-treated animals. Detailed morphological and immunohistochemical analysis did not show any differences in the macroscopic appearance, media thickness, proliferative activity, and apoptotic rate of VSMCs in aortic sections. Although these experiments only showed the condition in the adult animal, one can deduce that Crem also is not essential for development in general and especially in the formation of the contractile apparatus during mouse embryogenesis. Taken together, the basal increase in CRE-mediated gene expression in Crem^−/−^ mice particularly had no effect on the physiology of the vasculature and the quiescence of VSMCs.

Stimulation of VSMCs with Pdgf revealed an increase of CRE-mediated gene expression in the Crem^−/−^ VSMCs which was completely blunted in the Crem^+/+^ cells. This indicates that Crem is able to completely suppress the Pdgf-dependent CRE-mediated gene expression. Hence, conditions of elevated Pdgf release should generate an increased CRE-mediated gene expression in VSMCs with possible consequences for cellular physiology. We tested this hypothesis in vivo in the pathophysiological models of atherosclerotic plaque development in Crem × ApoE^−/−^ mice and the ligation of the carotid artery, which are associated with increased Pdgf production [47, 34, 6]. Despite Crem^−/−^ × *ApoE* ^−/−^ mice showing a slight increase of plaque burden after 30 weeks of high-fat diet, a detailed analysis of plague development revealed no differences between Crem^−/−^ × ApoE^−/−^ and Crem^+/+^ × ApoE^−/−^ mice. Ligation of the carotid artery in Crem^+/+^ mice was associated with a marked downregulation of *Crem* mRNA levels, supporting the relevance of Crem in this context. Accordingly, the complete inactivation of Crem led to a severe increase of neointima formation in the Crem^−/−^ mice associated with an increased proliferation rate of VSMCs in the media. This corroborates to results of Herring et al. showing that the majority of neointima-forming cells are derived from differentiated medial smooth muscle cells [12]. Although endothelium-mediated effects could not be excluded in this context, levels of Icam and Vcam reflecting endothelium activation via inflammatory mediators [43] showed no differences between the genotypes. Primary VSMCs of Crem^−/−^ mice exhibited a higher amount of proliferative cells after stimulation with Pdgf. Therefore, the increased neointima formation could be explained mainly by the elevated capacity of Crem^−/−^ VSMCs to proliferate. These findings fit well with results in rats suggesting that an adenoviral expression of the Crem isoform Icer inhibits VSMC proliferation and at least contributes to the effects of Crem in vasculature [28].

In primary Pdgf-stimulated Crem^−/−^ VSMCs, we found an upregulation of the Pdgf receptor, alpha polypeptide (*Pdgfra*) compared to Crem^+/+^ controls. Pdgf signaling via Pdgf receptors (Pdgfrs) plays an important role in the formation of the neointima after vascular injury, and administration of a Pdgfra antibody reduces neointima development in an allograft model in rats [34, 24]. Furthermore, Pdgfra levels increase during aging associated with an increase of proliferative response and increased neointima formation after wire injury [45]. Watson et al. showed that Pdgfra levels were inversely regulated to the Creb content in SMCs [46]. Since short Crem isoforms, acting as transcriptional repressors, can be rapidly induced via activated Creb/CRE-mediated signaling cascades [26, 38], Pdgfra content could be inversely regulated via an activator (Creb)-induced inhibitor (Crem) regulatory loop.

Moreover, cyclophilin A (*Ppia*) mRNA was increased in Crem^−/−^ VSMCs and is therefore associated with a higher proliferation rate. Since Ppia is a possible target gene of Creb [48], it might be activated by the inactivation of the repressor Crem. Ppia overexpression in VSMCs increases neointima formation and is involved in VSMC migration and proliferation as well as inflammatory cell accumulation [36], corroborating with our observations. Interestingly, we observed an upregulation of the regulator of G-protein signaling 5 (*Rgs5*) mRNA. The regulator of G-protein signaling 5 is a highly and differentially expressed member of the G-protein signaling (Rgs) proteins in the arterial smooth muscle. In a model of β_2_-adrenoceptor overexpression or after isoproterenol treatment, Rgs5 is upregulated in atria [16]. This links the expression of Rgs5 to cAMP-dependent signaling, and indeed, we found an upregulation of *Rgs5* in the aortae of Crem^−/−^ mice, which exhibit an increased cAMP-dependent gene expression. The role of Rgs5 in the vasculature is not understood in detail so far. Rgs5 is downregulated under Pdgf stimulation, and this downregulation was linked to an overall activation of smooth muscle cells associated with an activation of G_qα_/G_iα_ signaling, increased migration, hypertrophic response, and vascular remodeling [10]. Cho et al. showed that Rgs5 is upregulated during maturation and acts as a potent GTPase of G_qα_/G_iα_ signaling [4]. Arnold et al. reported an increased abundance of Rgs5 in growing collateral arterioles during arteriogenesis and a 70 % decreased neointima formation in Rgs5-deficient mice. They suggested that Rgs5 shifts G_αq/11_ signaling towards a G_α12/13_-mediated Rho kinase-dependent SMC activation [1] and Rho kinase inhibition was found to decrease neointima formation [39]. However, the specific roles of, e.g., Rgs5, Pdgfra, and Ppia and their possible mutual interdependencies need further evaluation.

In conclusion, Crem^−/−^ mice represent a valuable genetic mouse model to study the functional consequences of increased CRE-mediated transcriptional activation in vasculature. Crem is a potent repressor of Pdgf-CRE-mediated signaling associated with inhibition of proliferation in VSMCs. Moreover, our experiments show that Crem is an important regulator of gene expression under pathophysiological conditions, associated with increased Pdgf release, like vascular injury.

## Electronic supplementary material

Below is the link to the electronic supplementary material.

ESM 1 (DOCX 507 kb)
