# Supplementary material for: Transcription factor cAMP response element modulator (Crem) restrains Pdgf-dependent proliferation of vascular smooth muscle cells in mice
Source: Pflugers Arch. 2014 Nov 27;467(10):2165–77. doi: 10.1007/s00424-014-1652-6 (PMC4564437; doi:10.1007/s00424-014-1652-6)
Supplement: Supplementary file 1 — (DOCX 507 kb) [file 424_2014_1652_MOESM1_ESM.docx]

**Supplement: Material and Methods**

*Cell Culture.* Primary aortic smooth muscle cells (VSMCs) were isolated from adult Crem^-/-^ and Crem^+/+^ mice (aged 17-19 weeks) by the explant method. The explanted and cleaned aortae were cut into small pieces and the tissue mush was transferred to sterile culture flasks and allowed to dry for 5-10 min. Culture medium was added and cell growth could be detected after 7-10 days. Cells were subcultured with trypsin (0.05 %) / EDTA (0.022 %; PAA, Cölbe, Germany). For experiments VSMCs of passage 2 were used. Only for determination of the CRE activity in non-stimulated and forskolin-treated VSMCs (Fig 1 A) the VSMCs were obtained by enzymatic digestion[[1](#_ENREF_1)]. Briefly, the thoracic aorta was excised, separated from the connective tissue and placed in sterile ice-cold isolation medium (in mmol/l: 60 NaCl, 85 L-glutamic acid, 5.6 KCl, 2.0 MgCl_2_, 10 HEPES, pH 7.4). Four to five aortae were cut into small pieces and digested for 30-45 min at 37 °C in isolation medium containing 0.7 mg/ml papain, 1 mg/ml albumin, 1 mg/ml 1,4-dithiothreitol, followed by a centrifugation at 800 g and a second digestion for 20-30 min at 37 °C (1 mg/ml collagenase, 1 mg/ml hyaluronidase and 1 mg/ml albumin in isolation medium). Cells were collected by centrifugation, singularized by a cell strainer and counted. Cell culture was established plating 10.000 cells/cm^2^ on a 12-well polystyrene cell culture dish containing DMEM medium (Gibco/Invitrogen, Karlsruhe, Germany) supplemented with 20 % FCS (Gibco/Invitrogen) and 1 % Pen/Strep (Gibco/Invitrogen) at 37 °C in a humidified atmosphere with 5 % CO_2_. These cells were used for experiments after 7-8 days.

*Real Time analysis of VSMC proliferation rate by impedance measurements.* VSMCs were seeded on E-Plates 16 plates in a density of 10000 cells per well and measured in a RTCA DP Analyzer (ACEA Biosciences Inc., San Diego, USA). Cell were left in the wells to attach for 24 hours, after medium exchange (DMEM without serum) cells starved for 24 hours followed by a stimulation with the platelet-derived growth factor (Pdgf-BB, 7.5 ng/ml). The obtained cell index was normalized to the time point before Pdgf administration and the difference between Pdgf and untreated controls was calculated of each curve to determine the maximum cell index and the time to the maximum.

*Transfection.* To evaluate CRE-transcriptional activity, primary isolated aortic VSMCs were transiently cotransfected with a firefly luciferase reporter gene construct under the control of tetramerized CREs[[2](#_ENREF_2),[3](#_ENREF_3)] and a second plasmid expressing *Renilla reniformis* luciferase (pRL-CMV) under the control of the CMV early gene promoter (Promega, Mannheim, Germany) to normalize the transfection efficiency afterwards. Cells were transiently transfected using Lipofectamine 2000 (Invitrogen) according to the manufacturers instructions, mixing 0.95 µg of firefly luciferase reporter gene construct with 0.05 µg pRL-CMV and 1.5 µl Lipofectamine 2000. After 2.5 h, cells were washed and fed with complete medium. Initial experiments (not shown) revealed a maximum effect of stimulation with forskolin (1 μmol/l), a direct receptor-independent activator of the adenylyl cyclase, after 12 h. Hence, the experiments of forskolin stimulation were conducted at this time point. VSMCs were stimulated with forskolin (3x10^-8^ – 3x10^-5^ mol/l) or with DMSO (1x10^-3^ mol/l) as a solvent control. Stimulation with 8-(4-Chlorophenylthio)guanosine-3',5'-cyclic monophosphate (8pCPT-cGMP; **Biolog Life Science Institute, Bremen, Germany),** a cGMP analogon which stimulates the cGMP-dependent protein kinase (PKG), was carried out at a concentration of 10^-4^ M. Stimulation of VSMCs with Nitroso-N-acetylpenicillamin (SNAP), a nitric oxide donator was carried out at 10^-4^ M and Pdgf-BB was added at a concentration of 7.5 ng/ml after incubation with serum-free medium for 24 h. Then cell extracts were prepared and luciferase activity was measured using a luminometer and the Dual luciferase detection kit according to the manufacturer’s instructions (Promega, Mannheim, Germany). *Renilla reniformis* luciferase was used as an internal control to normalize the expression of the firefly luciferase reporter gene.

*Tissue and VSMC treatment for histological and immunohistochemical analysis.* Excised aortae or carotids were fixed overnight in neutral buffered formaldehyde (4 %) and, embedded in paraffin before 5 µm thick sections were prepared from each vessel on glass slides. Aortic sections were dewaxed in xylene, rehydrated in graded alcohols and routinely stained with hematoxylin-eosin. Diameter of aortic media was measured eight times rotating in a 45° degree angle around the cross-sectioned aorta. For analysis of neointima formation, carotids were stained with Resorcin-Fuchsin and differentiated with 3 % acid alcohol for visualization of internal elastic lamina, followed by a Nuclear Fast Red staining differentiated with 95 % ethanol (Waldeck, Division Chroma, Münster, Germany). For documentation of proliferative activity and apoptosis VSMCs were cultured in chamber slides and fixed in neutral buffered formaldehyde (4

%) for 20 min.

*Aortic plaque formation and serum lipids*. For analysis of atherosclerotic plaque development animals from the Crem^-/-^ strain were bred with ApoE^-/-^ (B6.129P2-Apoe^tm1Unc^/J, The Jackson Laboratory, Bar Habor, USA) mice. Breeding of offspring mice was continued for more than 5 generations to ensure a uniform genetic background. The Crem^-/-^ x ApoE^-/-^ and Crem^+/+^ x ApoE^-/-^ mice were fed with a high fat diet (EF R/M, TD88137 mod.; Ssniff, Soest, Germany) for 20-40 weeks beginning at 8 weeks of age. After atherogenic diet mice were sacrificed and serum lipid profiles (Centrum für Laboratoriumsmedizin, University Hospital Münster, Münster, Germany) and atherosclerotic lesions in the aorta were analyzed. Measurement of triglycerides, high- and low-density lipoprotein (HDL; LDL) and cholesterol revealed no differences between the genotypes (supplemental Figure S3). Hearts with the aortic root were isolated, fixed in neutral buffered formaldehyde (10 %) for at least 24 h and macrophage content was determined in aortic root sections using anti-Mac2 antibody (1:400 Cedarlane). Plaque size at the aortic root was determined using ImageJ software (1.37v , NIH). For quantification of plaque formation aortae were mounted on microscopic slides using aqueous mounting medium (Dako, Carpinteria, California, USA) scanned and analyzed with Image-PRO Analyzer (Media Cybernetics, Inc, Bethesda, Maryland, USA).

*Immunofluorescence imaging*. Isolated VSMCs were fixed by 5 min incubation with methanol, treated with Triton X-100 (0.25 %) and washed with PBS. Deparaffinized aortic sections were boiled in 10 mmol/l citrate solution (pH 6.0) for ten minutes. Cells or sections were blocked in PBS substituted with 2 % goat serum (Sigma–Aldrich) for 1 h, followed by treatment with an antibody against smooth muscle myosin heavy chain (Myh11; Abcam ab683,) 1:200 or Von Willebrand factor (Vwf Dako A0082) 1:200 or a none antibody control over night in blocking solution. After washing (at all times 3 x 10 min in PBS), probes were incubated with the secondary antibody AlexaFluor 594 (AlexaFluor® 594, Alexa-Fluor A11032, Goat Anti-Mouse IgG, Life Technologies) 1:500 in PBS for 1 h at room temperature. After washing nuclei were stained by 4',6-diamidino-2-phenylindole (DAPI, 10 pg/ml in distilled water) and mounted (Fluorescence mounting medium, Dako Deutschland GmbH, Hamburg). Fluorescence was detected using a fluorescence microscope (Ti-E Eclipse, Nikon GmbH, Düsseldorf). For analysis of proliferation, slides of VSMCs were treated with Triton X-100 (0.2 %) while deparaffinized carotid and aortic sections were boiled in 10 mmol/l citrate solution (pH 6.0) for ten minutes. After washing VSMCs or sections three times with PBS, followed by blocking with goat serum (0.2 %) for 1 h, incubation with primary antibodies Ki-67, Vcam1 or Icam1 (Thermo Fisher Scientific, Dreieich, Germany) was conducted for 1 h at room temperature. After washing with PBS, sections were incubated for 1 h with a secondary antibody Alexa-Fluor 488 (goat-anti-rabbit IgG) or Alexa-Fluor 594 (Life Technologies). Afterwards cell nuclei were stained with 4',6'-diamidino-2-phenylindole (DAPI) and mounted on a glass slide. In case of double immunofluorescence staining the Ki-67/Alexa-Fluor 488 specific staining was followed by washing with PBS and incubation with unconjuncated goat-anti-mouse-Fab fragments (100 µg/ml in PBS; Dianova Gmbh, Hamburg, Germany) for 1 hour. Afterwards the slides were washed and incubated with an alpha smooth muscle actin antibody (Acta2 United States Biological), washed again, then the secondary antibody Alexa 594 was added. After washing with PBS the slides were stained with DAPI and mounted, specificity of fluorescence staining was proved by a control without primary antibodies.

DNA fragmentation as a marker for apoptosis was detected by terminal uridine deoxynucleotidyl transferase dUTP nick end labeling (DeadEnd Fluorometric TUNEL System, Promega, Mannheim, Germany), according to the manufacturer’s instructions. Nuclei were stained with DAPI. The microscope slides were digitized using a high-resolution camera (DFC320, Leica Microsystems, Wetzlar Germany) attached to a fluorescence microscope (DMLB, Leica Microsystems, Wetzlar Germany). Quantitative analysis under standardized calibrated magnification was performed using a computer-based software system (Image Pro Plus 7.0 Media Cybernetics Inc., Bethesda, USA).

*Microarray.* Total RNA was extracted from primary VSMCs of Crem^-/-^ and *Crem^+/+^* mice with the use of RNA preparation kit (ZR RNA MicroPrep, Zymo Research, Freiburg, Germany). For generation of biotinylated, amplified cRNA a TotalPrep™-96 RNA Amplification Kit (**Life Technologies GmbH**, Darmstadt, Germany) was used according to the manufacturer’s instructions. An amount of 850 ng cRNA was hybridized on MouseRef-6 v2 Expressions BeadChips (Illumina, San Diego, USA) and chips were scanned on a Illumina BeadStation 500 and analyzed with BeadStudio Data (Illumina).

*Quantitative Real-Time Polymerase Chain Reaction (RT-PCR)*. Total RNA (0.5 µg) was randomly reversely transcribed into cDNA using the First Strand cDNA Synthesis Kit for RT-PCR AMV (Roche, Mannheim, Germany). The RT-PCR was carried out using a LightCycler 2.0 System (Roche) and the detection was performed by measuring the binding of the fluorescence dye SYBR Green I to double-stranded cDNA at 530 nm (QuantiFast SYBR Green PCR Kit; Qiagen). Primers (**Life Technologies GmbH**, Darmstadt, Germany) were designed using Primer3 (http://frodo.wi.mit.edu/;[[5](#_ENREF_5)]) with standard configuration except: “Mispriming Library” was set to “Rodent and Simple” and “Product Size” was limited to 100-300 base pairs.

Primer sequences were as follows: angiotensin II, type I receptor-associated protein (*Agtrap*) 5’-CTGATGACCTGGGTTCGAGT-3’ (forward), 5’-CCATCGAGGTTCCACTATTCT­C­-3’ (reverse); Rho GTPase activating protein 12 (*Arhgap12*), 5’-TGGGGAACAATTTGTGTGTG-3’ (forward), 5’-AGAGCCCTAAAGCACACTGC-3’ (reverse); fibroblast growth factor 18 (*Fgf18*) 5’-ATGAAGCGTTACCCCAAGG-3’ (forward), 5’-CTTTGTCATGGGGTTCAGGT-3’ (reverse); hypoxanthine guanine phosphoribosyl transferase (*Hprt*) 5’-GGAGTCCTGTTGATGTTGCCAGTA-3’ (forward), 5’-GGGACGCAG­CAACT­GACA­TTT­CTA-3’ (reverse); platelet derived growth factor receptor, alpha polypeptide (*Pdgfra*) 5’-GGCACGTTAACTGTTGCACT-3’ (forward), 5’-GGGGTATCTGGAAGCCATCT-3’ (reverse); cyclophilin A (*Ppia*) 5’-TTATCTGCACTGCCAAGACTGA-3’ 5’-GGGGAATGAGGAAAATATGGAA-3’;regulator of G-protein signaling 5 (*Rgs5*) 5’-GGGTGAGAAGGGTA­GGAAGG-3’ (forward), 5’-CATTTGGCTTTTCCTTT­GG­A-3’ (reverse); TAF12 RNA polymerase II, TATA box binding protein (TBP)-associated factor (*Taf12*) 5’-CACAGGGTGCATAGCTGAGA-3’ (forward) 5’-GGCA­GAG­AAA­GTGCTC­CAAC-3’ (reverse) and tyrosine 3-monooxygenase/tryptophan 5-monooxygenase activation protein, zeta polypeptide (*Ywhaz*) 5’-AG­CA­GGCAG­AGC­G­A­­TATGATGACA-3’ (forward) 5’-TCCCTGCTCAGT­GA­CAG­A­C­T­­TCAT-3’ (reverse). The PCR reactions were set up in microcapillary tubes in a volume of 20 µl. The reaction components were 2 µl undiluted cDNA, 10 µl QuantiFast SYBR Green PCR Master Mix, 4 µl H_2_O and 2 µl for each primer (10 pM). Reactions were incubated at 95 °C for 5 min followed by 50 cycles at 95 °C for 5 s, 60 °C for 15 s and 72 °C for 10 s. Relative levels of particular cDNAs were determined with the help of LightCycler software (version 3.5) with appropriate calibration curves obtained with different amounts of control cDNA. Crossing points were determined by the using the second derivative method. On completion of the PCR amplification, a melting curve analysis was performed. Relative quantification was performed by calculating relative expression ratios using *Hprt* and *Ywhaz* RNA as reference genes and the relative expression software tool (REST© Version 2.07; see ref.[[4](#_ENREF_4),[6](#_ENREF_6)]). Random statistical analysis was performed with 10000 iterations.

**
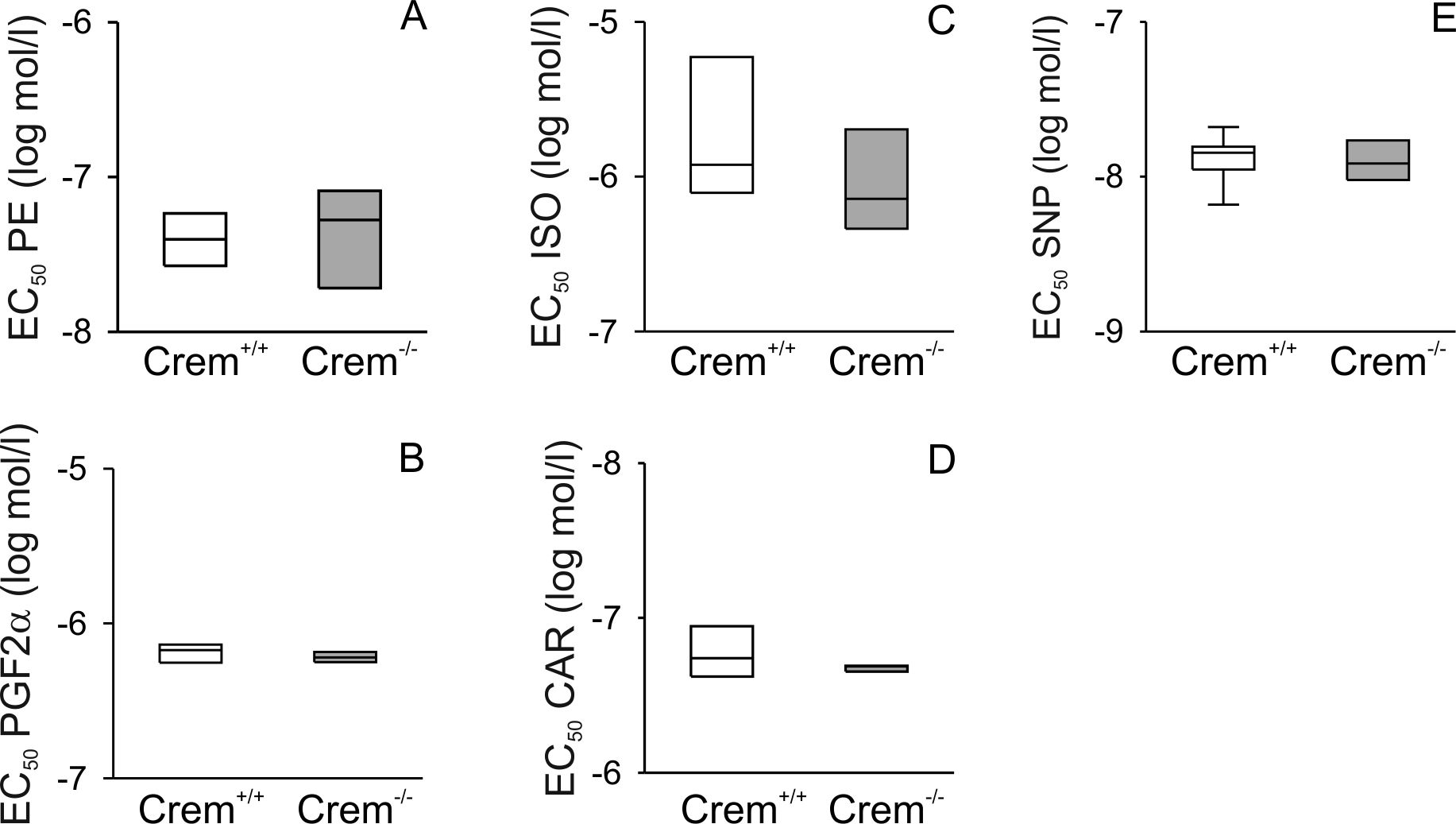
**

Supplemental Figure 1

**Supplemental Figure S1:** Calculated EC_50_ values of the drug-response curves were shown as Box and Whisker-Plots for Crem^+/+^ (white) and Crem^-/-^ (grey) mice. The vascular tone was measured in response to stimulation with the α-adrenoceptor agonist phenylephrine (A; PE; Crem^+/+^ n=6, Crem^-/-^ n=6) and the prostaglandin F_2α_ (B; PGF_2α_; Crem^+/+^ n=4, *Crem^-/-^* n=3). Relaxations were induced after pre-constriction with PE (1 µmol/l) by the β-adrenoceptor agonist isoproterenol (C; ISO; Crem^+/+^ n=4, Crem^-/-^ n=5), the muscarinic receptor agonist carbachol (D; CAR; Crem^+/+^ n=8, Crem^-/-^ n=5), and the nitric oxide donor sodium nitroprusside (E; SNP; Crem^+/+^ n=9, Crem^-/-^ n=6). Note that there were no significant differences between Crem^+/+^ and Crem^-/-^ mice in the maximum contraction or relaxation and in the EC_50_ values.


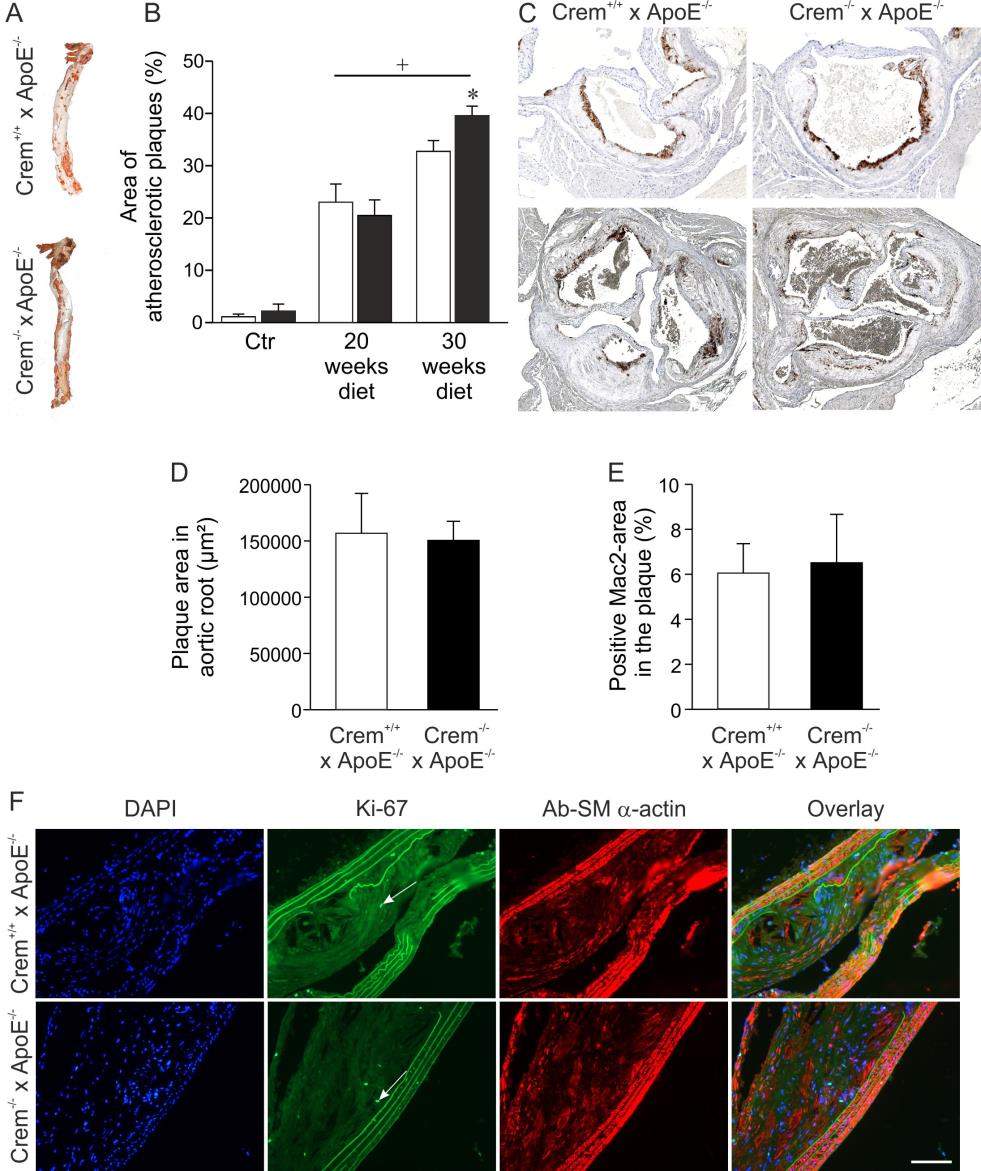


Supplemental Figure 2

**Supplemental Figure S2:** Development of atherosclerotic plaques of Crem^+/+^ x ApoE^-/-^ and Crem^-/-^ x ApoE^-/-^ mice after 20 to 40 weeks of high fat diet, respectively. (A) Photographs show Red-Oil-O stained aortae and (B) quantitative analysis of Red-Oil-O stained plaque area in the aortae from Crem^+/+^ x ApoE^-/-^ (white) and Crem^-/-^ x ApoE^-/-^ (black) mice after 20 (n=14-15) and 30 (n=14-15) weeks of feeding atherogenic high fat diet or standard diet as control (Ctr). Note the elevated plaque burden in the aortae of Crem^-/-^ x ApoE^-/-^ mice *p<0.05 vs. Crem^+/+^ x ApoE^-/-^ ; +p<0.05 vs. Ctr.. (C) Detection of Mac2-positive cells by immunohistochemistry in the aortic root. Quantitative analysis of plaque area (D) and Mac2 positive cells (E) in the aortic root revealed no differences between Crem^+/+^ x ApoE^-/-^ (white) and Crem^-/-^ x ApoE^-/-^ (black) mice after 40 weeks of high fat diet. (F) Detection of proliferating VSMCs in the cross sections of aortae of Crem^+/+^ x ApoE^-/-^ and Crem^-/-^ x ApoE^-/-^ mice. Photomicrographs show: the cell nuclei stained with DAPI, proliferating cells detected by a Ki-67 antibody (white arrows), visualization of VSMCs with a smooth muscle actin-specific antibody and the overlay. No difference in the amount of proliferating cells between the genotypes was detected (for details see results in the text). Scale bar=100 µm.


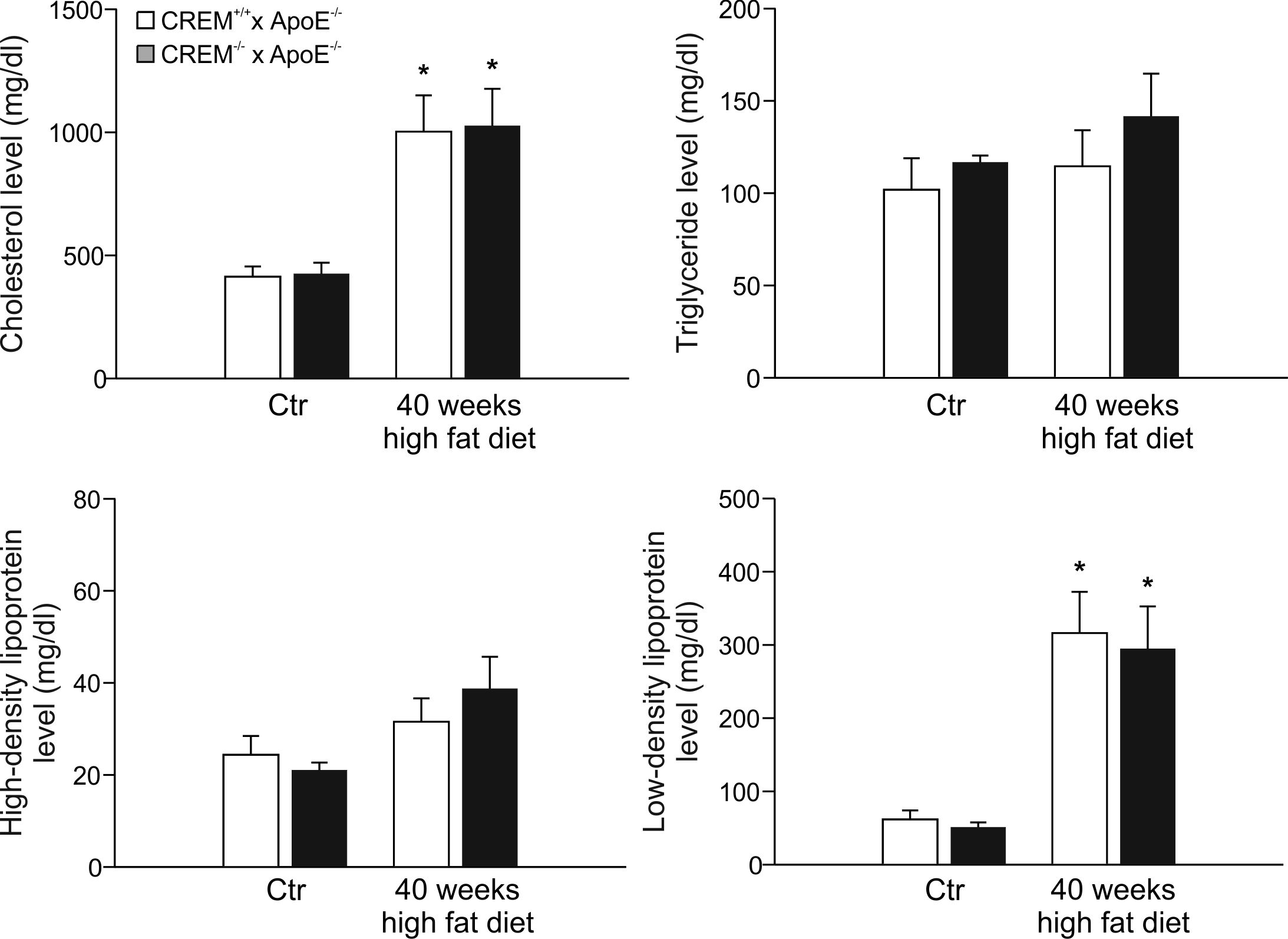


**Supplemental Figure S3:** Plasma lipid levels of Crem^+/+^ x ApoE^-/-^ (white) and Crem^-/-^ x ApoE^-/-^ (black) mice after 40 weeks of high fat (n=9-11) and standard (Ctr; n=5-6) diet. No significant differences between the genotypes were observed; *p<0.05 vs. Ctr.

Reference List

1. Kuhbandner S, Brummer S, Metzger D, Chambon P, Hofmann F, Feil R (2000) Temporally controlled somatic mutagenesis in smooth muscle. Genesis 28 (1):15-22. doi:10.1002/1526-968X(200009)28:1<15::AID-GENE20>3.0.CO;2-C

2. Muller FU, Neumann J, Schmitz W (2000) Transcriptional regulation by cAMP in the heart. Mol Cell Biochem 212 (1-2):11-17. doi:10.1023/A:1007176030884

3. Oetjen E, Diedrich T, Eggers A, Eckert B, Knepel W (1994) Distinct properties of the cAMP-responsive element of the rat insulin I gene. JBiolChem 269 (43):27036-27044. PMID:7929445

4. Pfaffl MW, Horgan GW, Dempfle L (2002) Relative expression software tool (REST) for group-wise comparison and statistical analysis of relative expression results in real-time PCR. Nucleic Acids Res 30 (9):e36. PMID:113859

5. Rozen S, Skaletsky H (2000) Primer3 on the WWW for general users and for biologist programmers. Methods in molecular biology 132:365-386. PMID: 10547847

6. Vandesompele J, De Preter K, Pattyn F, Poppe B, Van Roy N, De Paepe A, Speleman F (2002) Accurate normalization of real-time quantitative RT-PCR data by geometric averaging of multiple internal control genes. Genome biology 3 (7):RESEARCH0034. PMID:126239
